# Design, Simulation, and Experimental Characterization of a Superimposed Top- and Bottom-Gate Field-Emission Triode Fabricated Using a Post-CMOS MEMS Process

**DOI:** 10.3390/mi17091014

**Published:** 2026-08-27

**Authors:** Yu-Hsien Wu, You-Ting Chen, Ting-Wei Chang, Wen-Teng Chang

**Affiliations:** Department of Electrical Engineering, National University of Kaohsiung, Kaohsiung 81148, Taiwaneps91713@gmail.com (T.-W.C.)

**Keywords:** field emission triodes, dual gate, Fowler–Nordheim tunneling, gate field emission, electrical conductance, floating gate, gate biasing

## Abstract

This study presents a comprehensive experimental and theoretical investigation into dual-gate field-emission devices fabricated using a standard 0.35 µm CMOS-MEMS process. Two emitter configurations, the concave-tip and triangular-tip, are characterized, and their performance is rigorously analyzed using three-dimensional simulations based on Fowler–Nordheim emission theory. To account for discrepancies between initial designs and fabricated devices, the influence of critical geometric parameters, including tip apex radius, cathode-anode spacing, and tip sharpness, is systematically evaluated regarding emission current and threshold voltage. Compared to the floating-gate baseline (~38 V), dual-gate (DG) operation lowers the threshold voltage by ~70% (~10 V), enhances low-voltage emission over tenfold, and provides a 3.4-fold boost in differential output conductance. Simulation analysis indicates this improvement stems from enhanced electrostatic field distribution governed by the gates. Furthermore, the top gate, due to its proximity to the emitter tip relative to the bottom gate, provides superior control over emission current at lower operating voltages. Three-dimensional simulations corroborate these findings, revealing that minimizing both the tip radius and cathode-anode spacing substantially enhances tunneling electron flow. Additionally, gate voltage sweeps confirm that electron trajectories are effectively directed by electrostatic steering. These findings establish critical design guidelines for integrating field-emission devices into standard CMOS platforms, facilitating the development of on-chip electrostatically controlled electron sources for integrated vacuum microelectronics.

## 1. Introduction

Nanoscale field-emission electronics have emerged as a promising technology, benefiting from ballistic electron transport in vacuum/air channels and high tolerance to extreme operating conditions [1,2,3,4]. However, for field-emission triodes (FETs) to match the integration density and efficiency of modern semiconductor transistors, optimizing their electrostatic gate control is essential. While aggressive downscaling improves high-frequency and radio-frequency performance [5,6], efficient current modulation and low operating voltages rely heavily on the spatial arrangement of the gate relative to the emission tips [7,8,9,10,11,12,13,14,15]. Consequently, precise geometric optimization of this layout is required. In general, making the emitter sharper (i.e., with a smaller taper angle) and taller improves field-emission performance, leading to a lower turn-on field and a higher macroscopic field enhancement factor [16].

Among prevailing architectures, horizontal FETs offer superior compatibility with standard planar complementary metal-oxide-semiconductor (CMOS) technologies. By leveraging these mature processes, researchers can transition from specialized laboratory prototyping [8,11,17,18] to fabricating fully IC-compatible FETs on standard platforms [17]. Furthermore, horizontal FETs equipped with vertical top or bottom gates provide a strategic advantage by effectively modulating the current and electron trajectories [11,17,18,19,20,21,22]. Nevertheless, determining the optimal positioning and combination of these gates remains a critical design challenge.

While recent advancements have successfully demonstrated monolithic on-chip FETs integrated with passive components using a 0.35 µm CMOS-MEMS process [17], those studies predominantly focused on circuit-level integration and transient dynamics. A systematic investigation into how complex 3D geometries and asymmetric gate configurations influence the local electric field is still lacking. Because field emission is strictly governed by local field strength, precise 3D modeling is indispensable for evaluating the impact of tip curvature, sharpening angles, and cathode-to-anode spacing. While previous numerical studies demonstrate that emission current (I_emission_) can be optimized by tuning basic geometric parameters, such as making the emitter more slender near the apex [12], these specific configurations are not the primary focus of our present design. Furthermore, the nominal emission area of the tip still remains largely undetermined [23]. Consequently, it is only through detailed simulations incorporating specific parameters that this effective emission area can be reasonably approximated.

Regarding gate design, although preliminary simulations suggest that asymmetric dual-gate layouts can balance low capacitance with strong electrostatic control [11], a direct and systematic comparison between single-gate and dual-gate configurations is necessary to maximize modulation efficiency. Building upon previous demonstrations, the primary objective of this work is to elucidate how specific gate architectures (namely top-gate (TG), bottom-gate (BG), and combined dual-gate (DG) configurations) dictate the modulation efficiency and voltage requirements of sharp-tip FETs. Furthermore, by utilizing the radius-to-gap ratio, previous research has established a new empirical formula to better predict quantum tunneling and improve future nanoelectronic devices [15]. By systematically correlating 3D geometric simulations with experimental current-voltage characteristics, conductance measurements, and Fowler-Nordheim (F-N) analyses, we provide a comprehensive evaluation of gate-dependent device performance. Ultimately, this fundamental understanding establishes a critical foundation for optimizing the structural design of monolithic field-emission devices within standard IC platforms.

Building upon our previous work demonstrating the first monolithically integrated multi-gate field emission device [17], the present study utilizes a 0.35 µm CMOS-MEMS process to extend the single-gate architecture to a dual-gate configuration, specifically evaluating its impact on differential output conductance and electrostatic modulation. While modulating emission current via gate electrodes is a well-established concept [8,24,25], our multi-gate design is uniquely tailored to standard CMOS-MEMS foundry constraints. In this process, the poly1 layer serves as the standard CMOS gate, while the poly2 layer is typically employed for integrated resistors. Consequently, rather than using conventional overlapping structures, the electrodes are designed with a vertically staggered profile that complies strictly with foundry design rules. Furthermore, we employ 3D simulations to systematically analyze process-induced physical variations, specifically cathode-to-anode gap fluctuations and tip blunting from isotropic/anisotropic etching. Compared to traditional planar and 3D FED configurations, this process-compatible, staggered multi-gate architecture provides a novel platform for precise emission control while offering a realistic simulation framework to guide the design of fully integrated field emission microelectronics.

## 2. Device Design and Fabrication

### 2.1. Device Design

**Figure 1:**
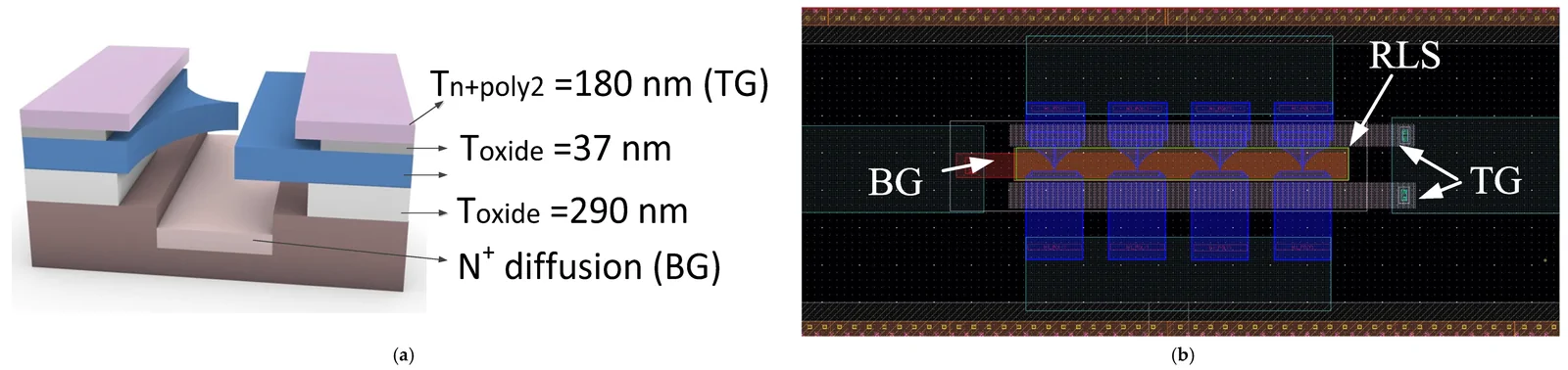
(**a**) 3D schematic illustrating the layer stack and thicknesses of the dual-gate field-emission triode (FET), featuring the n^+^ poly2 top gate (TG), the poly1 FET structure (C–A), and the n^+^ diffusion bottom gate (BG). The dual-gate (DG) FETs are simultaneously connected to both TG and BG, while the air channel is defined by anisotropic ion fluorine release etching and followed by isotropic Si etching. (**b**) Layout schematic: BG (red), two parallel TGs (grey), and release-layer region RLS (orange).

The FETs were fabricated at the Taiwan Semiconductor Research Institute (TSRI) using a 0.35 µm CMOS process integrated with a post-CMOS MEMS module. This standard IC technology node is widely adopted in industry and available through multi-project wafer (MPW) services. The structure employs a DG FET configuration (Figure 1a), in which the BG is implemented using an n^+^ diffusion region, while the TG is formed by an n^+^ poly2 layer. The cathode and anode (C–A) electrodes are realized in the poly1 layer. Although intrinsically semiconductors, both polycrystalline silicon layers (poly1 and poly2) are heavily doped in this standard process to function as conductors. The specific material properties are proprietary and extracted directly from the foundry’s Process Design Kit (PDK). The vertical spacing between the C–A and the TG is approximately 37 nm, which is significantly smaller than the 290 nm separation between the C–A and the BG across the oxide stack. This precise alignment is achieved through a well-controlled multi-project wafer (MPW) process. The air channel is created by vapor-phase HF, produced from CHF_3_ (or other fluorine-containing gases) in the plasma, which first etches the SiO_2_ interlayer dielectric, followed by radical fluorine etching of the silicon. As illustrated in Figure 1b, the top-view layout displays the BG, the two parallel TG electrodes, and the designated sacrificial-layer release (RLS) region. To investigate asymmetric emission between the two sides, the C–A electrodes are patterned with both tip and flat terminations. Furthermore, two distinct tip geometries are employed: a concave-tip FET (C-FET) and a triangular-tip FET (T-FET).

### 2.2. Gap Formation and Double-Gate FETs

**Figure 2:**
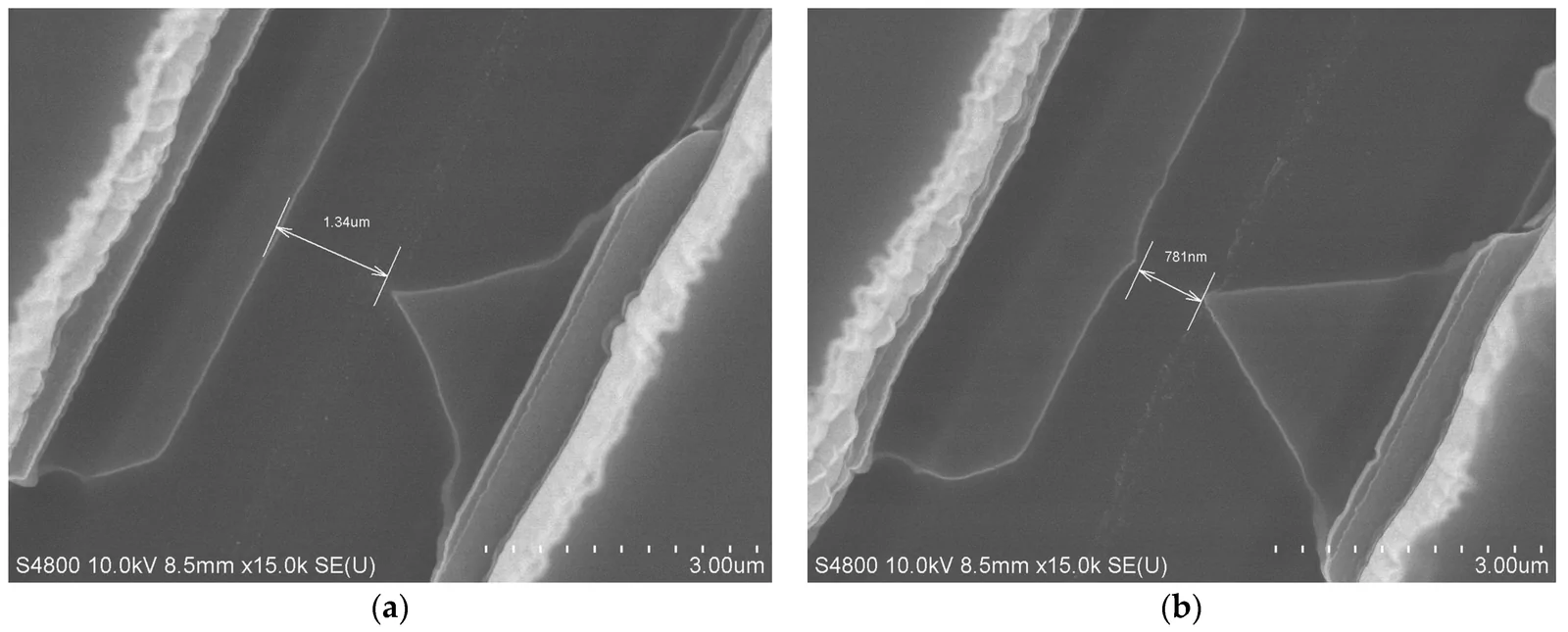
SEM images of (**a**) a concave tip-flat FET (C-FET) and (**b**) a triangular tip-flat FET (T-FET). Both configurations consist of four pairs of FETs.

The C–A distance in the current tip-to-flat FETs is larger than the intended layout value, primarily because polysilicon oxidation and its subsequent removal by ion fluorine, together with the resolution limits of the mature process node, effectively increase the gap. In addition, the opening gap is found to depend on the geometry of the polysilicon electrodes. The sharper C-FET, shown in Figure 2a, typically features a wider interelectrode spacing than the less sharp T-FET, shown in Figure 2b. Although the photomask targeted ~100 nm nanogaps, post-CMOS MEMS release etching inevitably expanded physical gaps to 1.34 µm (C-FET) and 781 nm (T-FET). Simulating 10–100 nm gaps provides design guidelines for future process optimization while quantifying scaling trends. Emitter geometry plays a critical role in device operation. Sharper emitter tips with smaller radii of curvature significantly reduce the turn-on and threshold electric fields, thereby lowering the required operating voltage, particularly when combined with shortened vacuum channels [8]. Motivated by this consideration, two emitter tip geometries were designed and compared.

Although the gap exceeds the mean free path of electrons in air, the strong local electric field remains highly sensitive to the tip apex radius [14]; measurable emission can still occur even when the current (I_CA_) is small. The Keysight B1500A semiconductor device parameter analyzer, with a current resolution on the order of 0.1 fA, is therefore well suited for capturing these ultra-low field-emission currents. In addition to BG-only and TG-only operation, a third type of DG FET is biased simultaneously through both TG and BG using a T-adapter, and the electrical conductance of the single-gate and DG configurations is evaluated by superimposing BG and TG bias, defined as the measured current divided by the applied voltage.

To evaluate device-to-device reproducibility, a total of 5 C-FET and 5 T-FET samples were characterized under identical testing conditions. Benefiting from the standardized CMOS-MEMS fabrication platform, the variation in turn-on voltage and emission current across different devices remained within a narrow margin of 15%. This variation is primarily attributed to relatively larger fluctuations in the cathode-anode gap spacing resulting from post-CMOS MEMS release etching, as well as localized tip apex blunting.

## 3. Modeling Setup

### 3.1. Modeling Framework and Fowler–Nordheim Emission Formulation

The geometry consists of three main parts: an emitter, a collector, and the surrounding air domain. COMSOL Multiphysics version 6.1 is employed to perform the numerical simulations and to compare different device geometries. In this framework, the electrostatics module computes the steady-state potential and electric field from the device geometry and charge distribution, yielding the local field enhancement at the tip and providing the field data required for the Fowler–Nordheim (F–N) emission boundary condition. Building on this solution, the charged particle tracing module solves the time-dependent motion of individual electrons in the calculated field to reconstruct their trajectories, thereby capturing transport and gate-induced deflection and enabling evaluation of the collected current and gate interception ratio. The local I_emission_ density is obtained based on the extended Murphy-Good (EMG) theory [1], as expressed in Equation (1)

(1) $$J_{EMG}=\frac{A_{FN}}{\emptyset }{\left(F_{local}\right)}^{2}e^{\left(-\nu \frac{\beta }{F_{local}}\right)}$$

where the $A_{FN}$ represents the first Fowler–Nordheim constant as defined in Equation (2), and β is the exponent coefficient defined in Equation (3), derived from electron mass m, reduced Planck constant $\bar{h}$, and elementary charge q.

(2) $$A_{FN}=\frac{q^{3}}{8\pi h}=1.541434\times 10^{-6}(A\cdot eV\cdot V^{-2}),$$

(3) $$\beta =B_{FN}\emptyset^{3/2},\;where\;B_{FN}=\frac{4\sqrt{2m}}{3\bar{h}q}=6.83\times 10^{9}\left(V\cdot m^{-1}\cdot {eV}^{-3/2}\right),$$

The total I_emission_, as defined in Equation (4), is calculated by integrating the local Fowler–Nordheim current density J_FN_ over the emitting surface. To achieve this, the surface is discretized into N mesh elements with effective areas ΔA_e_, and the total current is determined by summing the current contribution from each element, ensuring both the local and integrated values accurately reflect the field distribution. To ensure the numerical accuracy of the field-enhancement factor while avoiding high computational costs, the simulation domain must be appropriately sized [14]. Furthermore, while the extended Murphy-Good (EMG) theory serves as the primary framework in this work, recent literature notes that for extremely sharp emitters with tip radii of curvature below 20 nm, specialized high-curvature correction models, such as those proposed by Kyritsakis-Xanthakis [26] and Biswas-Ramachandran [27,28], are valuable for rigorously capturing image-charge effects and local field enhancements.

(4) $$I=\sum_{e=1}^{N}J_{EMG}(x_{e})\Delta A_{e}$$

It should be noted that the current I derived from Equation (4) represents the total emission current I_emission_ extracted directly from the cathode apex. In two-terminal configurations without gate electrodes, the total calculated emission current integrated at the cathode apex is assumed to be fully collected by the anode, so that I_CA_ is approximately equal to I_emission_ under ideal transport assumptions. In three-terminal gate-modulated configurations, the collected anode current (I_CA_) is estimated by coupling the electrostatic Fowler-Nordheim emission integration with particle-tracing collection fractions, where I_CA_ = I_emission_ × (1 − α) and α = I_gate_/I_emission_ represents the geometric electrostatic interception ratio rather than a fully self-consistent space-charge-limited transport current. The ratio of intercepted electrons is strictly governed by the applied gate bias and spatial electron trajectories.

**Figure 3:**
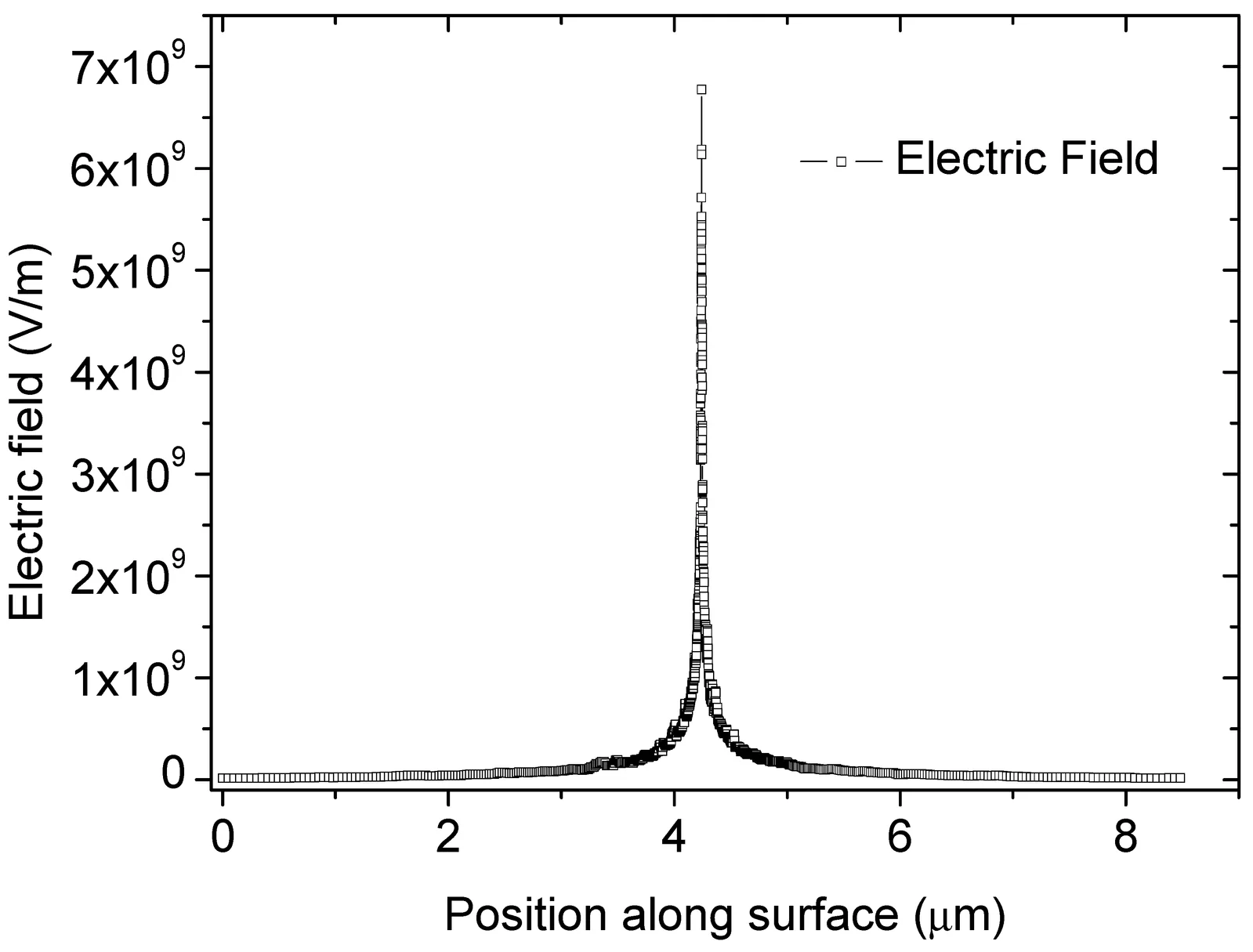
Local electric field distribution along the surface of the emitter tip, extracted from the 3D finite-element electrostatic simulation. The profile illustrates a sharp field concentration at the apex, reaching a peak value of approximately 6.8 × 10^9^ V/m which rapidly decays away from the apex.

Table 1 summarizes the parameters and symbols used in the Fowler-Nordheim (F-N) analysis. Under realistic operating conditions, the refined I_emission_ density based on the extended Murphy-Good (EMG) formulation incorporates image-charge corrections and local barrier fields [29]. Here, the local electric field (F_local_) at the emitter apex is directly resolved via 3D finite-element electrostatic simulations, naturally capturing the strong field enhancement induced by the sharp nanometric curvature without relying on crude constant approximations. As demonstrated by the arc-length field profile in Figure 3, the local electric field is intensely concentrated at the sharp apex, reaching a peak value of approximately 6.8 × 10^9^ V/m. Moving outward from the apex, the field decays rapidly, dropping to below 10% of its maximum value within a distance of just 0.1 μm along the emitter surface. Because the active emission region is strictly localized within this narrow nanometric domain, minor geometric modifications or truncations of the rear bulk structure do not disturb the surface charge density at the apex. This confirms that the local field enhancement is dictated by the nanoscale tip sharpness rather than the overall bulk geometry.

**Figure 4:**
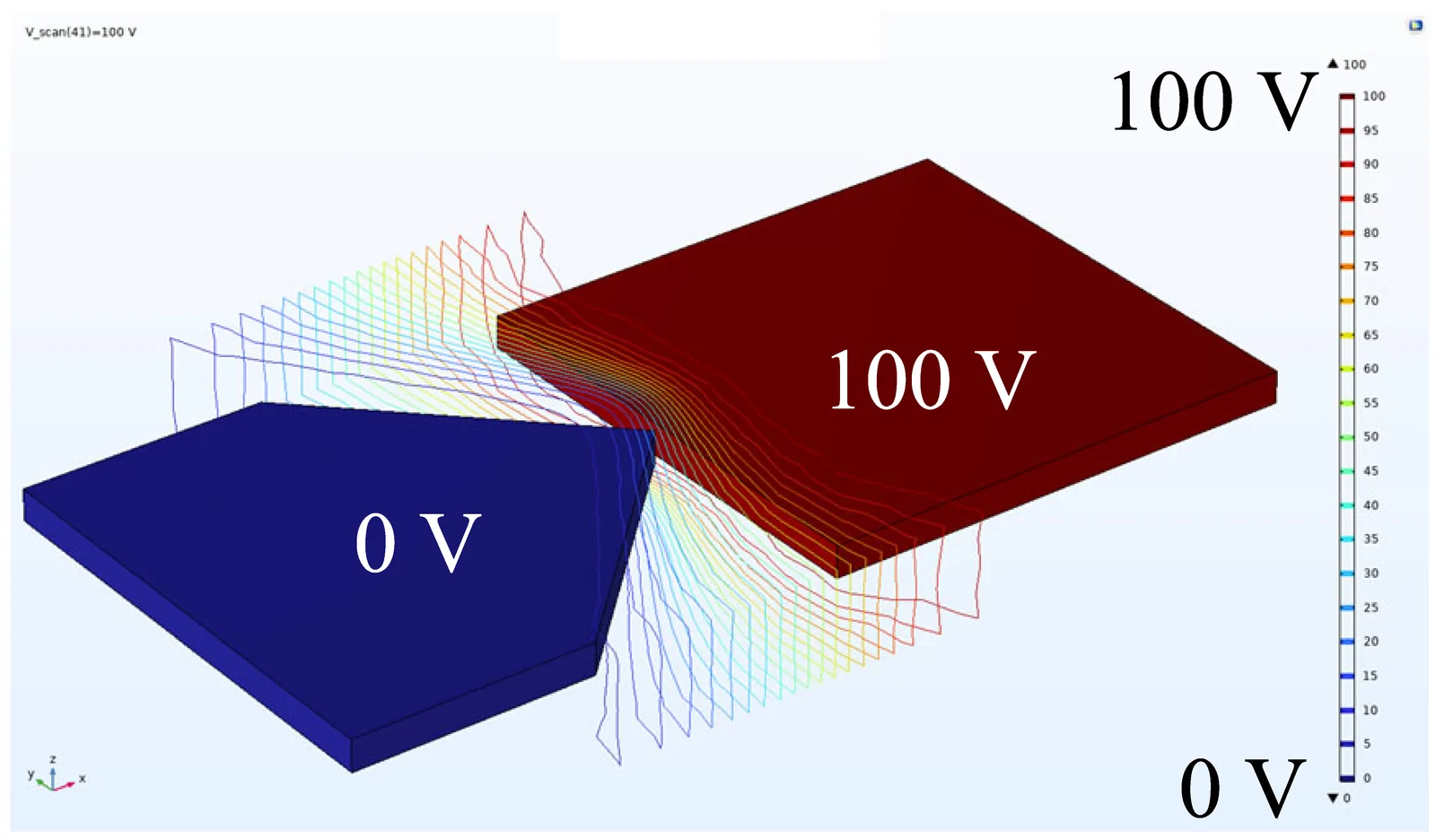
3D finite-element simulation of the electric potential (V) distribution from the tip emitter to the flat counter-electrode under an applied scan voltage of 100 V, in which the electric field (E) is the negative gradient of the electric potential, that is E = −∇V.

To model electron transport, Figure 4 presents the simulated 3D electric potential distribution and corresponding electron trajectories traversing from the sharp tip to the planar counter-electrode under an applied bias of 100 V. In these calculations, the dimensionless field parameter f_val_ scales F_local_ against the effective work function phi (4.28 eV for polysilicon), while the second Nordheim function ν = 1 − f_val_ + (1/6)f_val_·ln(f_val_) serves as the barrier-shape correction factor.

### 3.2. Numerical Modeling of Geometrical Optimization

#### 3.2.1. Tip Rounding

**Figure 5:**
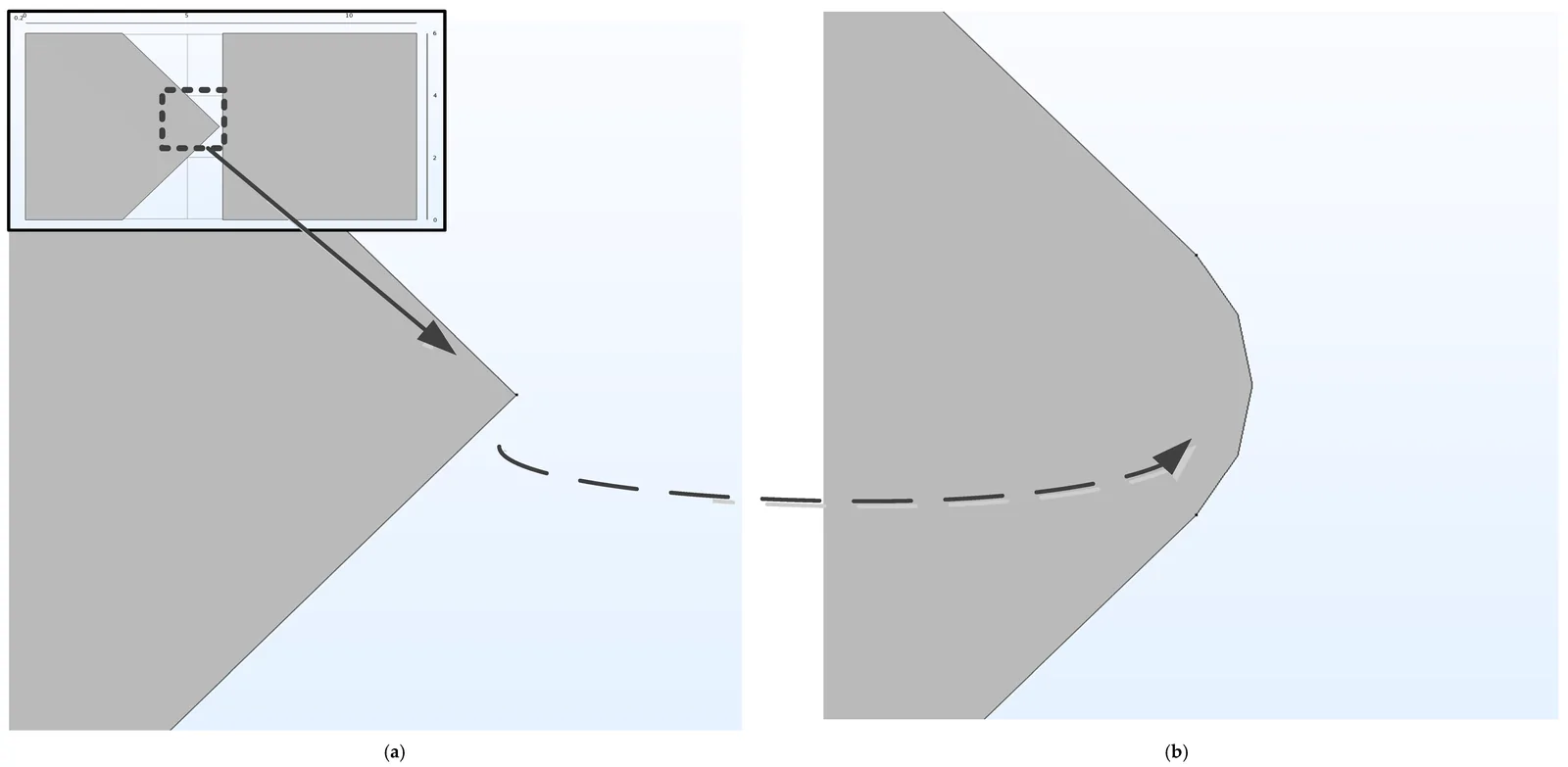
Schematic illustration of field-emission tip geometries: (**a**) an ideal sharp tip in a tip-to-flat cathode–anode configuration (inset) and (**b**) a representative blunted tip profile typically obtained after CMOS-MEMS fabrication. The dashed arrow indicates tip blunting during fabrication.

Although previous theoretical and experimental studies have confirmed that I_emission_ is highly sensitive to tip sharpness, silicon-based field emitters fabricated via standard CMOS MEMS processes exhibit relative resistance to tip passivation. Conversely, field emission devices featuring metal emitters (which cannot withstand high-temperature processing) are particularly susceptible to tip passivation [8]. When an extremely small inter-electrode gap is maintained between the cathode and anode, such passivation can severely degrade the field I_emission_ and overall device performance. Therefore, the primary motivation for this tip rounding simulation study is to systematically investigate the explicit impact of tip passivation on emission characteristics. Figure 5a depicts the intended sharp tip used in the design, while Figure 5b represents the practically achievable tip shape after fabrication, in which the apex is inevitably rounded. The emitter tip was rounded along the apex using a cylindrical fillet to better represent the actual physical geometry. As the fillet radius changes, the local current density changes accordingly. The impact of tip radius values of 5, 30, 50, and 90 nm on the current is investigated. The simulated material is polysilicon with a spacing of 100 nm.

#### 3.2.2. Influence of Tip Sharpness on Gap Geometry

**Figure 6:**
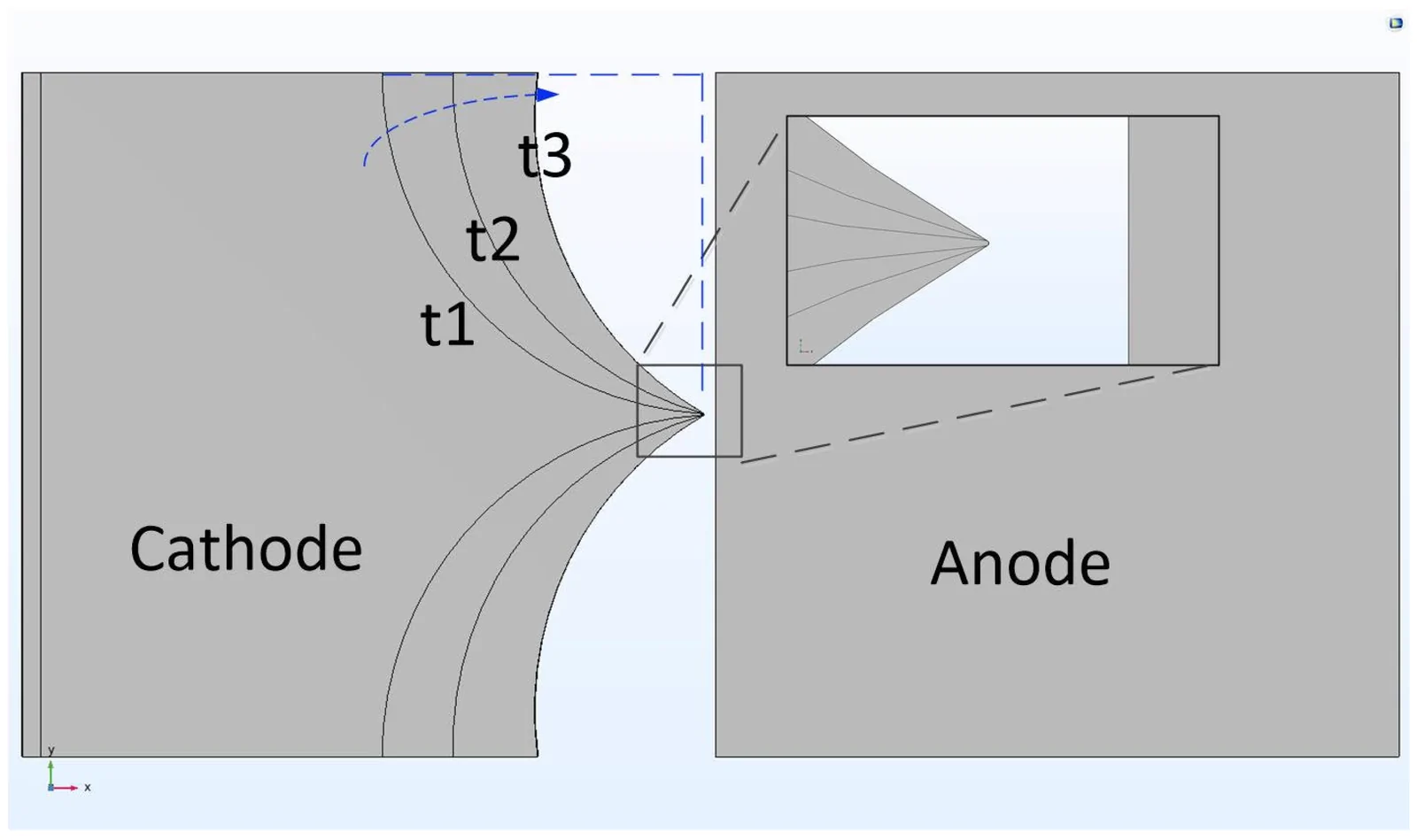
Schematic representation of the three tip-sharpness configurations: t1 (quarter-circle geometry producing the sharpest emission point), t2 (15°-rotated profile), and t3 (30°-rotated profile resulting in the flattest emission surface). The blue dashed curved arrow indicates the rotation from t1 to t3, and the black dashed lines denote the enlarged view of the tip region (inset).

Although the gap-distance dependence of field emission is well known, standard theoretical models rely on idealized boundaries that ignore practical fabrication constraints. In monolithically integrated CMOS MEMS field emitters, the inter-electrode gap distance is uniquely defined by sequential isotropic and anisotropic etching processes rather than a simple planar boundary. Consequently, conventional formulas cannot accurately evaluate the true electrostatic environment created by these process-defined gaps. Therefore, the primary motivation for simulating the gap geometry is to systematically quantify the exact relationship between the degree of gap reduction and the resulting I_emission_ within a realistic, process-driven framework. Three tip-sharpness configurations, denoted t1, t2, and t3 (Figure 6), are examined to study the influence of curved and rectangular tips as cathodes in this fabrication. In t1, a quarter circle with a radius equal to half of the square side (3 μm) is used. In t2, the same circle is rotated by 15° about its center, and in t3 it is rotated by 30° about the same center. As illustrated in the right-hand figure, t1 produces the sharpest tip, followed by t2, whereas t3 exhibits the flattest tip. To more accurately represent practical conditions, a 5 nm fillet radius is introduced at the tip. This modification causes a substantial tip recession in t1, increasing the originally designed 100 nm gap to 300 nm, and produces a similar effect in t2, where the gap increases by 20 nm. The gaps are subsequently corrected back to 100 nm.

#### 3.2.3. Impact of Cathode-Anode Distance in Air

In the current post-CMOS MEMS process, the actual gap between the cathode and anode can deviate significantly from its intended design value due to anisotropic SiO_2_ etching and isotropic silicon etching by fluorine radicals. To account for this variation, the resulting current is analyzed as a function of the actual separation between the silicon cathode and anode. To ensure precise spatial resolution during this analysis, the minimal mesh size is 4.2485 × 10^−4^ μm throughout the simulation. Because the electric field (E) is inversely proportional to the gap distance (d), expressed by the relationship E = β·V/d, reducing the separation inherently increases the field strength. Based on the Fowler–Nordheim relation in Equation (1), this reduction drives a pronounced surge in current density through both the quadratic increase in the electric field and the strong amplification of the exponential term. Furthermore, narrowing the gap can push regions that initially experienced sub-threshold electric fields above the emission threshold, yielding a substantial increase in the total I_CA_.

#### 3.2.4. Electron Projection with the Impact of Gate

For the electrical boundary conditions, the cathode (electron emitter) is fixed at 0 V, while the anode voltage is swept up to 100 V. During the simulations, the mesh on the cylindrical surface is locally refined to improve accuracy and ensure that the calculated current closely represents realistic device behavior.

The structural configuration employs a double-gate design with dimensions consistent with the fabricated devices, providing additional control over the electric-field distribution and modulation of the tunneling current. The resulting I–V characteristics under various gate biases are systematically analyzed and compared with experimental measurements, and the agreement between simulated and measured current–voltage curves is used to validate both the measurement methodology and the modeling framework.

## 4. Experimental Characterization of Field Emission

### 4.1. Effect of Floating and Biased Gates in Curved FETs (C-FET) and Triangular FETs (T-FET)

**Figure 7:**
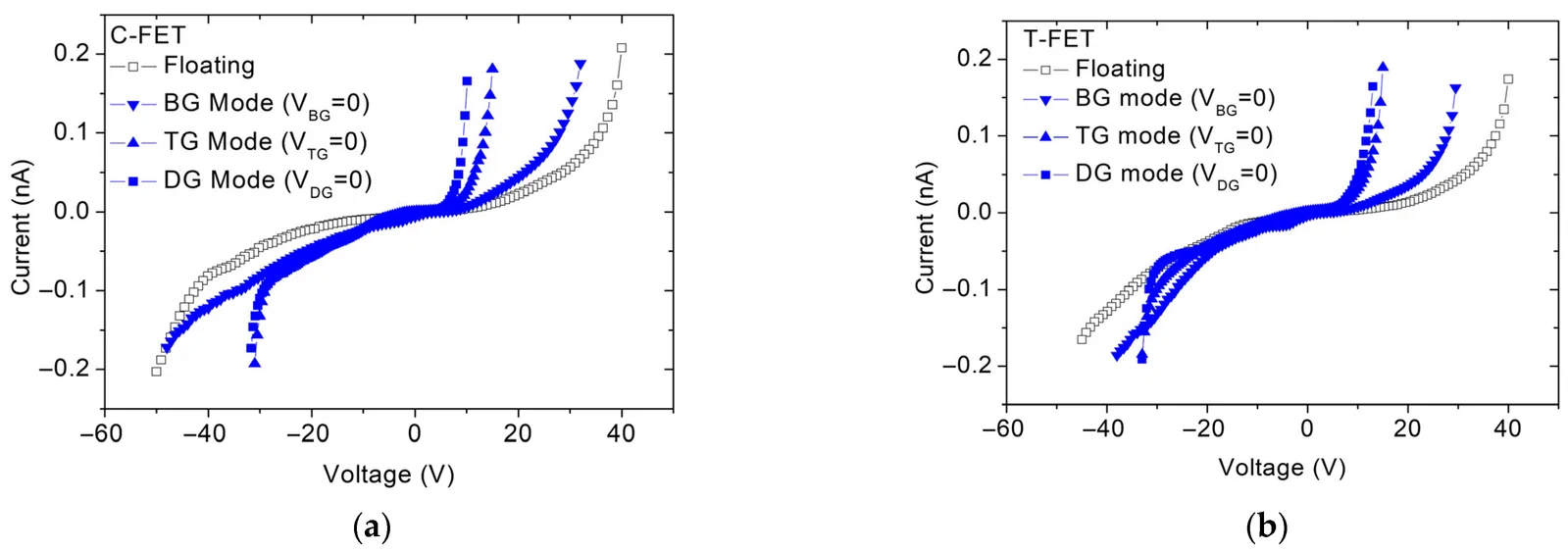
Current–voltage (I_CA_–V_CA_) characteristics for C-FET (**a**) and T-FET; (**b**) under floating-gate and biased-gate configurations, including BG (V_BG_ = 0 V), TG (V_TG_ = 0 V), and DG (V_DG_ = 0 V). For single-gate measurements (BG or TG), the unmeasured gate electrode is left floating. All biased conditions are referenced to the grounded cathode tip.

Figure 7a,b present the I–V characteristics of the C-FET and T-FET, respectively, under both floating (unconnected third probe) and biased configurations of the BG, TG, and DG, with all gate biases set to 0 V. The results show that the threshold voltage in the biased-gate configurations is lower than in the floating case for achieving the same I_CA_. Specifically, relative to the floating-gate baseline (~38 V to achieve ~0.15 nA), dual-gate (DG) operation reduces the threshold voltage by ~70% (down to ~10 V) and increases low-voltage I_CA_ by >1 order of magnitude. This occurs because anchoring the gate electrodes to a defined ground potential (V_G_ = 0 V) establishes a fixed electrostatic boundary (providing a lower relative potential when a positive voltage is applied to the opposing electrode), which significantly enhances the local electric field gradient near the tip compared to an ungrounded, floating gate.

The higher current observed in the DG C-FET at a given tip–flat voltage is attributed to the modified electron trajectories and field distribution produced by the dual-gate configuration, which enables a larger fraction of emitted electrons to reach the flat electrode, despite the steeper FN slope compared with the BG device. Among the biased cases, the BG, located farther from the anode than the TG, provides weaker local field modulation at a given voltage, whereas the DG, combining the capacitive coupling of both BG and TG, demands the lowest voltage. For a given FET, the DG exhibits the lowest threshold voltage, followed by the TG and then the BG, consistent with the gates modifying the surrounding field distribution and with the TG being closer to the emitter apex than the BG.

The plots also indicate that emission from the tip (with a positive voltage applied to the flat electrode) is smoother than emission from the flat (with a negative voltage applied). This difference is especially evident when the BG, TG, and DG are biased at 0 V compared with the floating condition, implying that emission from the flat surface is more strongly influenced by the presence of a grounded gate boundary. Specifically, under these negative cathode–anode bias conditions, the minor measurable reverse current is attributed to reverse field emission, wherein the localized sharp structural edges and sidewall corners of the flat (anode/collector) electrode experience sufficient field enhancement to emit electrons toward the cathode tip under inverted bias polarity.

### 4.2. Combined Differential Output Conductance of BG and TG Acting as DG in a FET

**Figure 8:**
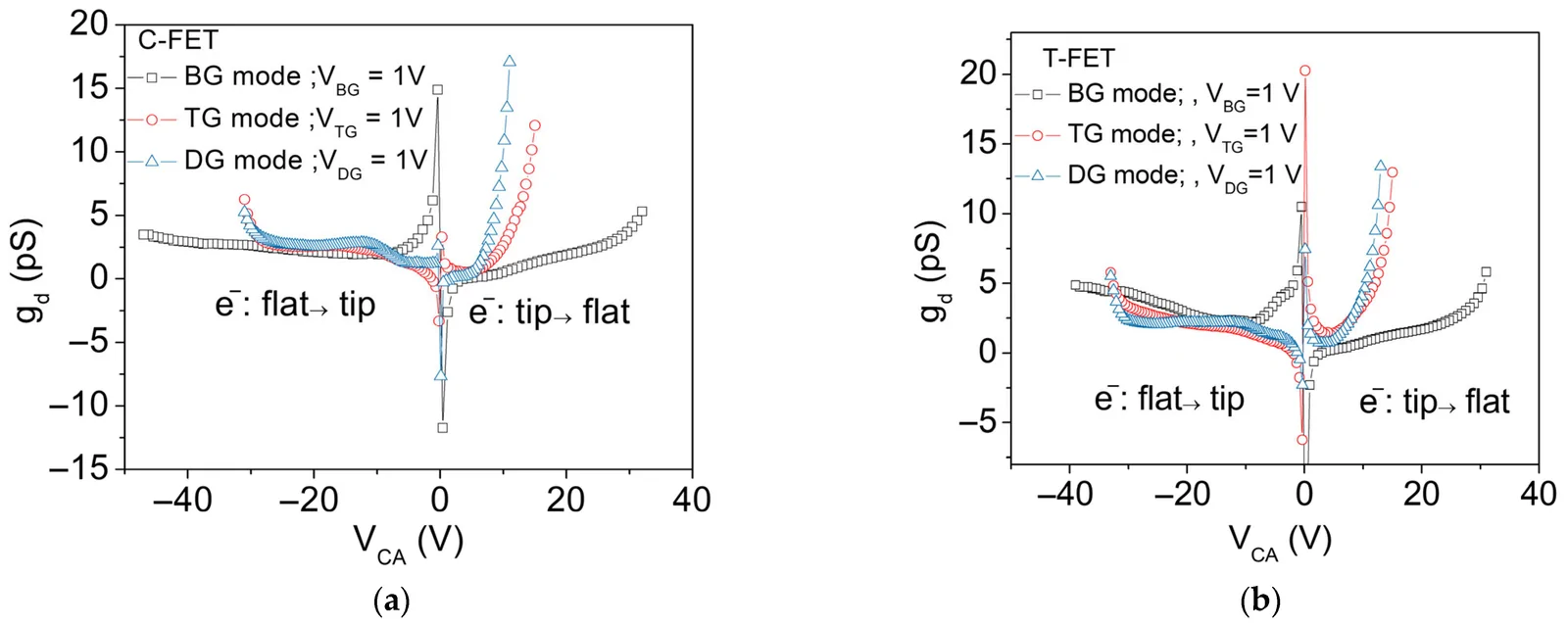
Differential output conductance (g_d_) versus collector-cathode voltage (V_CA_) under a gate bias of V_G_ = 1 V for BG, TG, and DG configurations in (**a**) C-FET and (**b**) T-FET. The g_d_ is calculated as the derivative of current with respect to V_CA_, referenced to the grounded emitting electrode. For single-gate modes (BG or TG), the unmeasured gate is left floating.

Based on the I-V characteristics under V_G_ = 1 V (Figure 7a,b), the differential output conductance (g_d_ = ${dI}_{CA}/{dV}_{CA}$)-voltage curves for both C-FET and T-FET demonstrate that operating the tip as the electron emitter under positive bias (V_CA_ > 0) yields significantly higher g_d_ than operating the flat region under negative bias (Figure 8a,b). At identical bias (V_CA_~10–15 V, V_G_ = 1 V), dual-gate (DG) operation significantly enhances g_d_. In C-FETs, DG mode peaks at ~17 pS (Figure 8a), providing a 3.4-fold boost over the single bottom-gate (BG, ~5 pS) and a 1.4-fold increase over the single top-gate (TG, ~12 pS) configurations. T-FETs similarly exhibit strong gate modulation, reaching peak values of 13–20 pS under biased-gate modes (Figure 8b), confirming the superior modulation efficiency of the dual-gate architecture at low operating voltages.

Across all gate configurations, the C-FET requires a lower driving voltage than the T-FET despite its substantially longer transport distance (1.34 µm vs. 781 nm), confirming that local field enhancement at the sharper tip apex dominates voltage reduction. Similar trends occur at V_G_ = −1 V. Furthermore, under forward electron flow (tip to flat), the combined g_d_ of BG and TG approximately equals that of DG. Under reverse bias, this superposition relationship becomes less consistent, likely due to lower electron collection efficiency at the tip anode and enhanced gate leakage current (I_gate_).

### 4.3. Fowler–Nordheim Analysis of C-FET Under Gate Bias

**Figure 9:**
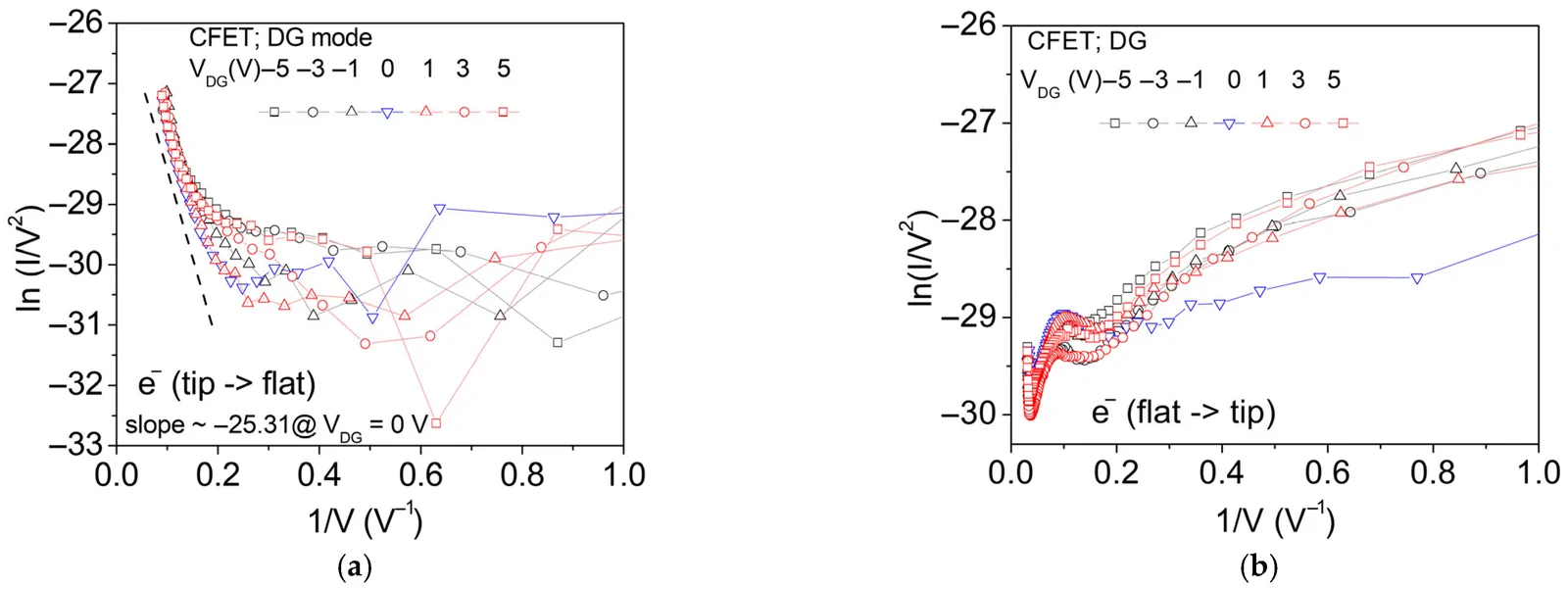
FN plots of the C-FET in DG mode under various gate biases (V_DG_ = −5, −3, −1, 0, 1, 3, and 5 V) for (**a**) forward emission with the tip acting as the cathode (V_CA_ > 0) and (**b**) reverse emission with the flat region acting as the cathode (V_CA_ < 0). All driving voltages V_CA_ are referenced to the grounded emitting electrode. The dashed line indicates a linear F–N fit slope of approximately −25.31 at V_DG_ = 0 V.

**Figure 10:**
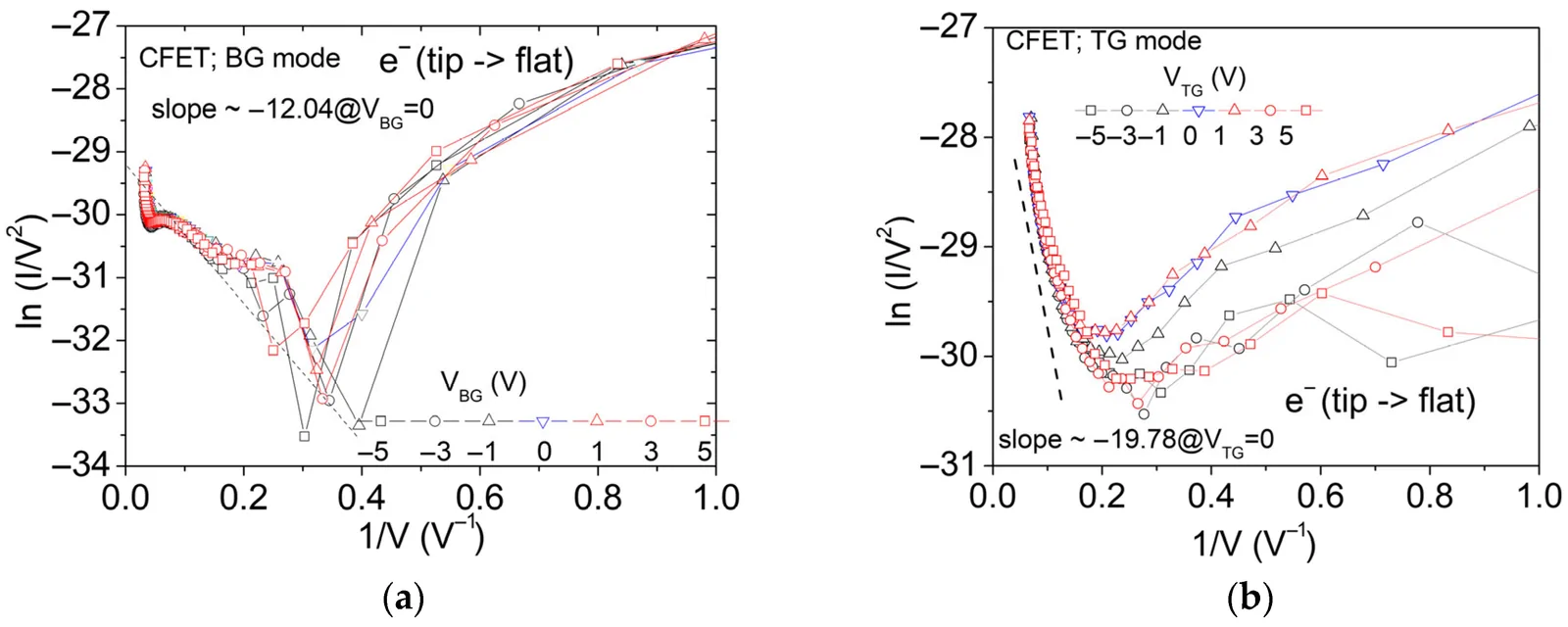
FN plots of the C-FET under various gate biases (V_BG_ and V_TG_ = −5, −3, −1, 0, 1, 3, and 5 V) in (**a**) BG mode and (**b**) TG mode, with the tip acting as the emitting cathode (V_CA_). Applied voltages V_CA_ are referenced to the grounded cathode tip, and the unmeasured gate electrode in each single-gate mode is left floating. The dashed line in (**b**) represents a linear F–N fit slope of approximately −12.04 at V_BG_ = 0 V in (**a**) and −19.78 at V_TG_ = 0 V in (**b**).

FN theory describes quantum tunneling emission and relates the emission current density to the local electric field. The FN plots for positive bias (electron flow from tip to flat) in the C-FET under gate voltages V_DG_ = −5, −3, −1, 0, 1, 3, and 5 V are shown in Figure 9a, while those for negative bias (electron flow from flat to tip) are shown in Figure 9b. The FN contribution is more pronounced for $V_{CA}$ > 0 than for $V_{CA}$ < 0, consistent with the g_d_ behavior in Figure 8a,b. For positive bias, the DG configuration in Figure 9a and the BG and TG configurations in Figure 10a,b all exhibit an approximately linear relationship between $ln(\frac{I}{V^{2}})$ and 1/V in the high-electric-field region (1/V < 0.2), confirming that the emission is governed by Fowler–Nordheim tunneling. Among them, the DG configuration exhibits the steepest FN slope (−25.31 ± 0.48 V), indicating a larger effective emission barrier or a smaller tip field-enhancement factor than the TG (−19.78 ± 0.41 V) and BG (−12.04 ± 0.33 V) configurations (Figure 10a,b). As summarized in Table 2, the TG configuration exhibits a larger FN slope magnitude than the BG case due to its thinner dielectric layer (37 nm vs. 290 nm) and stronger localized spatial potential modification near the tip apex. The steep FN slope in the DG mode (−25.31 ± 0.48 V) indicates a modified electrostatic barrier profile and dynamic field confinement around the emitter tip during tunneling. High coefficients of determination (R^2^ > 0.986) confirm that field emission across all gate configurations is governed by Fowler–Nordheim tunneling in the high-field regime.

It should be emphasized that the FN slope itself characterizes the local barrier geometry and electron emission physics, rather than serving as a direct metric for overall gate-modulation efficiency. Instead, the true gate-modulation efficiency of the triode is quantified through the transfer characteristics (I_CA_–V_G_) discussed in Section 4.4, including the gate voltage shift required to modulate the I_CA_ and the effective transconductance under gate biasing.

### 4.4. Gate Voltage Dependent Field Emission

**Figure 11:**
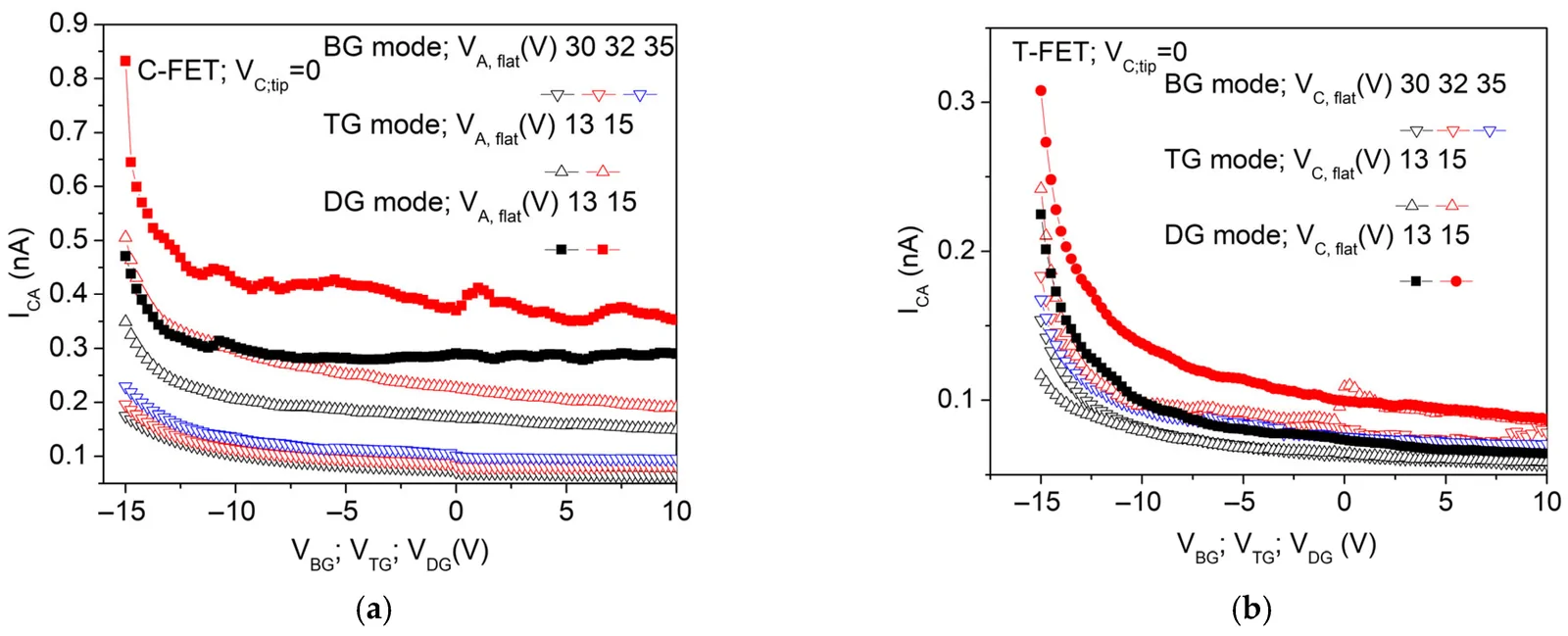
I_CA_ as a function of gate bias across different V_CA_ operating ranges, comparing BG, TG, and DG modes in (**a**) C-FET and (**b**) T-FET. All applied gate and anode (collector) voltages are referenced to the grounded cathode tip. For single-gate configurations (BG or TG), the unmeasured gate electrode is left floating.

Figure 11a,b present $I_{CA}$ as a function of $V_{G}$ for different $V_{CA}$ ranges of BG, TG, and DG for the C-FET and the T-FET, respectively. The $I_{CA}$ of both configurations decrease with $V_{G}\;(V_{BG},{\;V}_{TG},{\;V}_{DG})$ moving to the positive side. In both conditions, the emitter tip serves as the grounded cathode (0 V). Rather than acting as a secondary electron source, a negatively biased gate (V_G_ < 0 V) modulates the I_CA_ primarily by altering the local electrostatic potential profile near the emitter apex and deflecting electron transport pathways.

Additionally, the current in the C-FET remains higher than that in the T-FET. For the BG configuration, a much higher control voltage $V_{CA}$ is required to suppress the electrical current in both device types. In the DG case, most of the reduction in $I_{CA}$ is governed by the TG, because the distance from the tip to the TG is shorter than that from the tip to the BG. In summary, the top gate provides more efficient modulation of the I_emission_ than the bottom gate, enabling a lower control voltage to suppress $I_{CA}$ due to its shorter separation from the tip.

## 5. Simulated Field Emission Characteristics

### 5.1. Influence of Tip Apex Curvature on Field Emission

**Figure 12:**
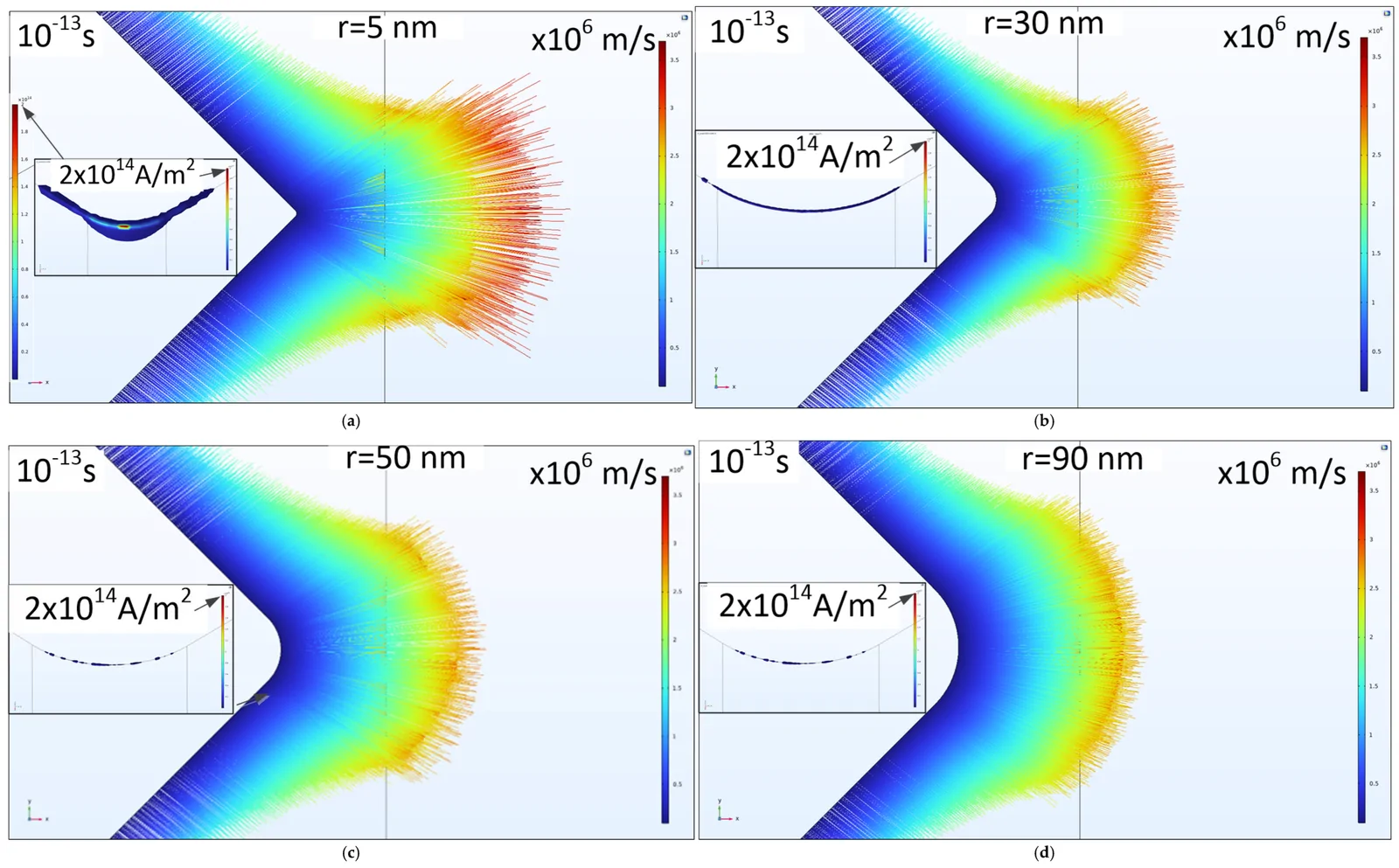
Simulated electron trajectories and velocity distributions (m/s) for tip radii of (**a**) 5 nm, (**b**) 30 nm, (**c**) 50 nm, and (**d**) 90 nm. Electrons are emitted from a 0 V polysilicon tip across a 100 nm air gap toward a 100 V planar counter electrode. Insets show the local current-density distributions at the emitter apex.

**Figure 13:**
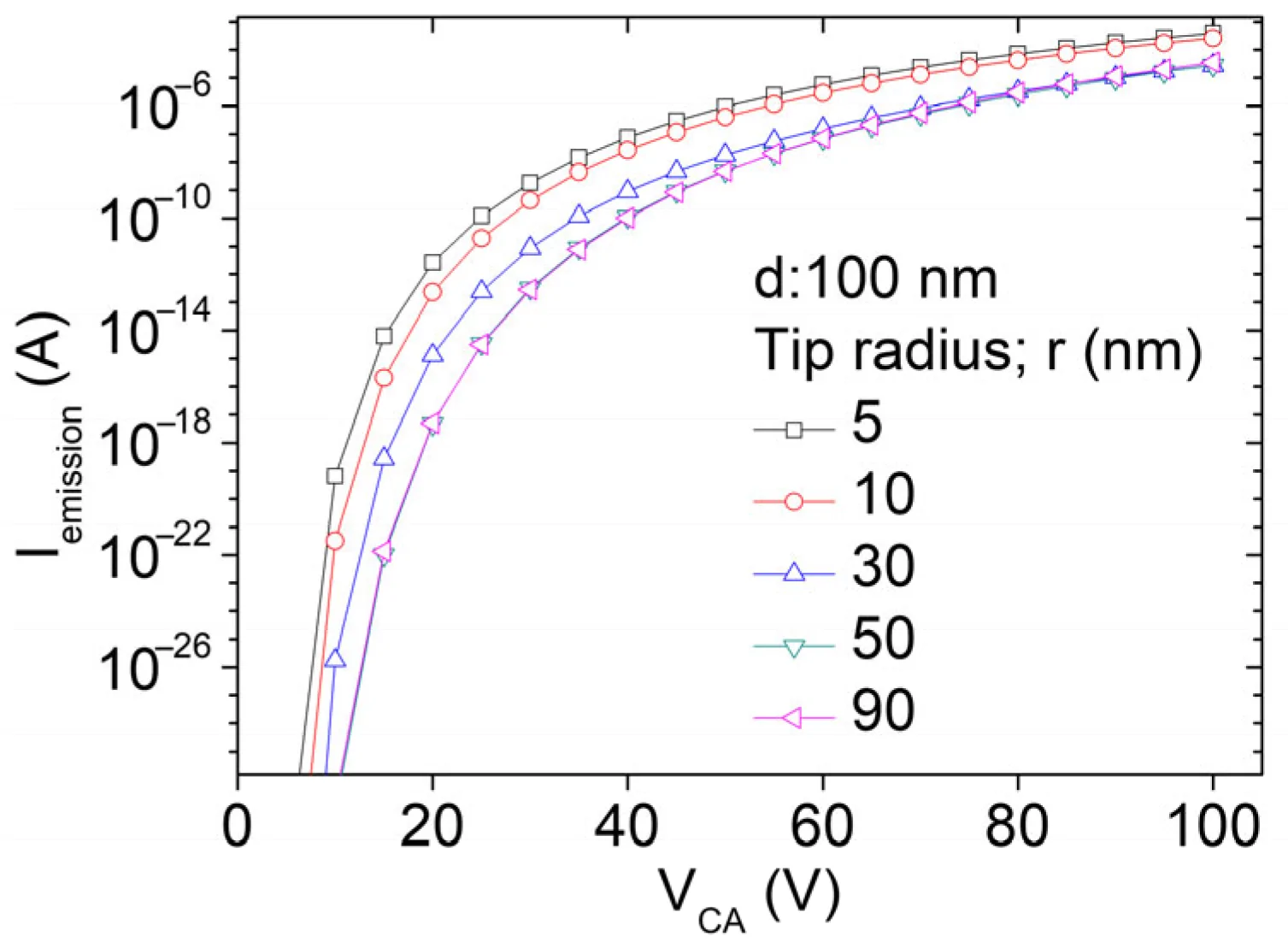
Simulated field I_emission_ as a function of applied voltage for tip-to-flat emitter–collector structures with tip radii (r) of 5, 10, 30, 50, and 90 nm. All simulations assume a 100 nm air gap (d) between the emitter and the counter electrode.

Comparisons between experimentally observed profiles and ideal sharp design layouts frequently reveal fabrication-induced tip blunting. Typically caused by over etching or thermal reflow, this phenomenon results in varying degrees of apex rounding, particularly in metal structures fabricated via standard CMOS-compatible MEMS processes [30]. To evaluate the impact of this blunting, Figure 12a–d present top-view simulations of electron trajectories and velocity distributions for tip radii of 5, 30, 50, and 90 nm. The insets illustrate the effective current density for different sharpness, depicted using a color scale with a maximum value of 2 × 10^14^ A/m^2^. Crucially, these insets provide a direct visual analysis of the region with significant local electric field enhancement, highlighting the effective spatial zone over which high-density electron emission is concentrated. As shown, the blunter emitter (e.g., inset of Figure 12d) exhibits a drastically reduced region of localized field enhancement compared to the sharper emitter (e.g., inset of Figure 12a). Note that the main trajectory plots in Figure 12a–d visualize the continuous velocity field across the emitter, where side-surface electrons exhibit significantly lower velocities due to weaker local electric fields. In contrast, the actual emission probability follows the Fowler-Nordheim theory, which is exponentially localized at the high-field apex as explicitly demonstrated by the current density insets. Therefore, while side trajectories map the spatial electrostatic field, their contribution to the total I_emission_ is negligible. As corroborated by Figure 13, these simulations demonstrate that the total I_emission_ is acutely sensitive to tip acuity. For instance, under an applied bias of 40 V, a sharp 5 nm tip generates an I_emission_ one to two orders of magnitude higher than that of a rounded 90 nm tip. This pronounced performance degradation in blunter emitters arises because the diminished electric-field concentration at larger radii significantly reduces the local current density, lowers the electron velocity near the apex, and ultimately restricts the effective field-enhancement region. 

### 5.2. Cathode-Anode Spacing Dependencies in Air

Because electric field strength is approximately inversely proportional to the distance between the emitting and collecting electrodes, reducing the cathode-anode spacing substantially increases the local electric field at the emitter surface. Under these conditions, both FN and direct tunneling current densities rise alongside the electric field, though FN tunneling exhibits a significantly stronger enhancement as the surface field grows larger.

**Figure 14:**
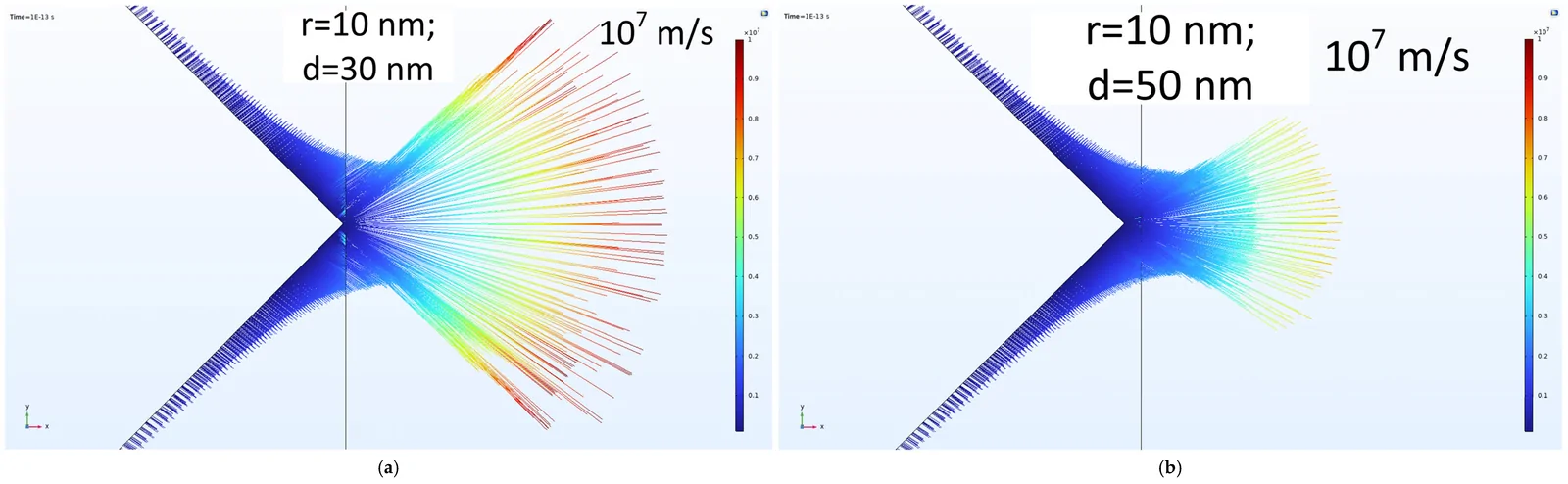

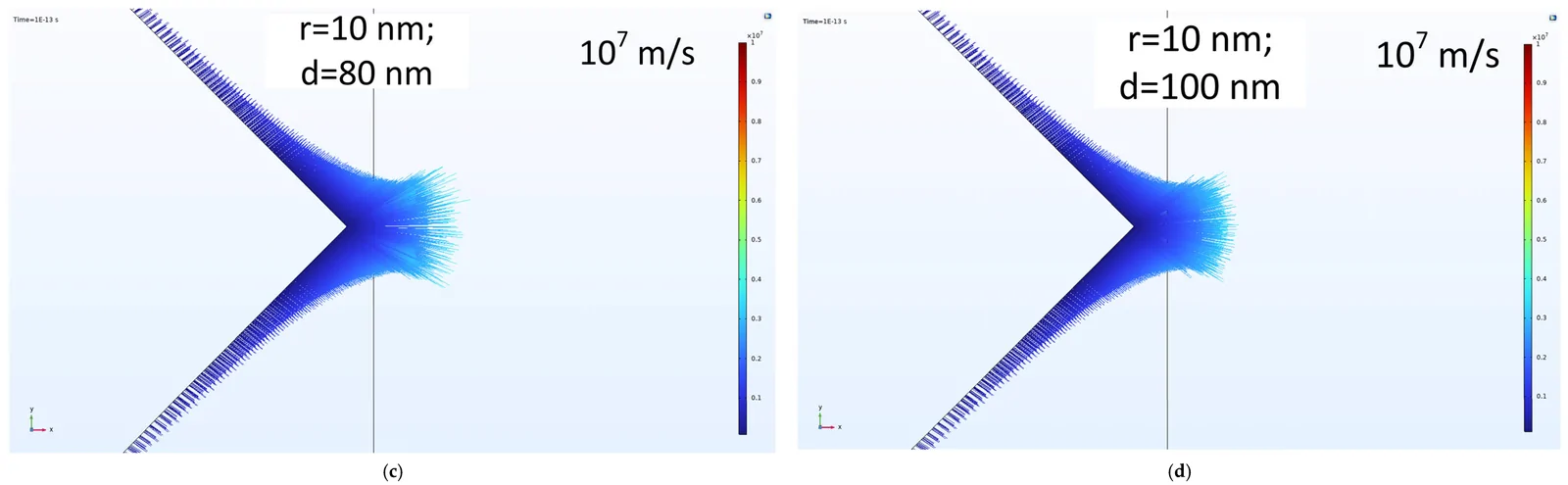
(**a**–**d**) Simulated electron trajectories from the tip emitter to the flat electrode for cathode–anode spacings (**d**) of 30, 50, 80, and 100 nm, respectively.

**Figure 15:**
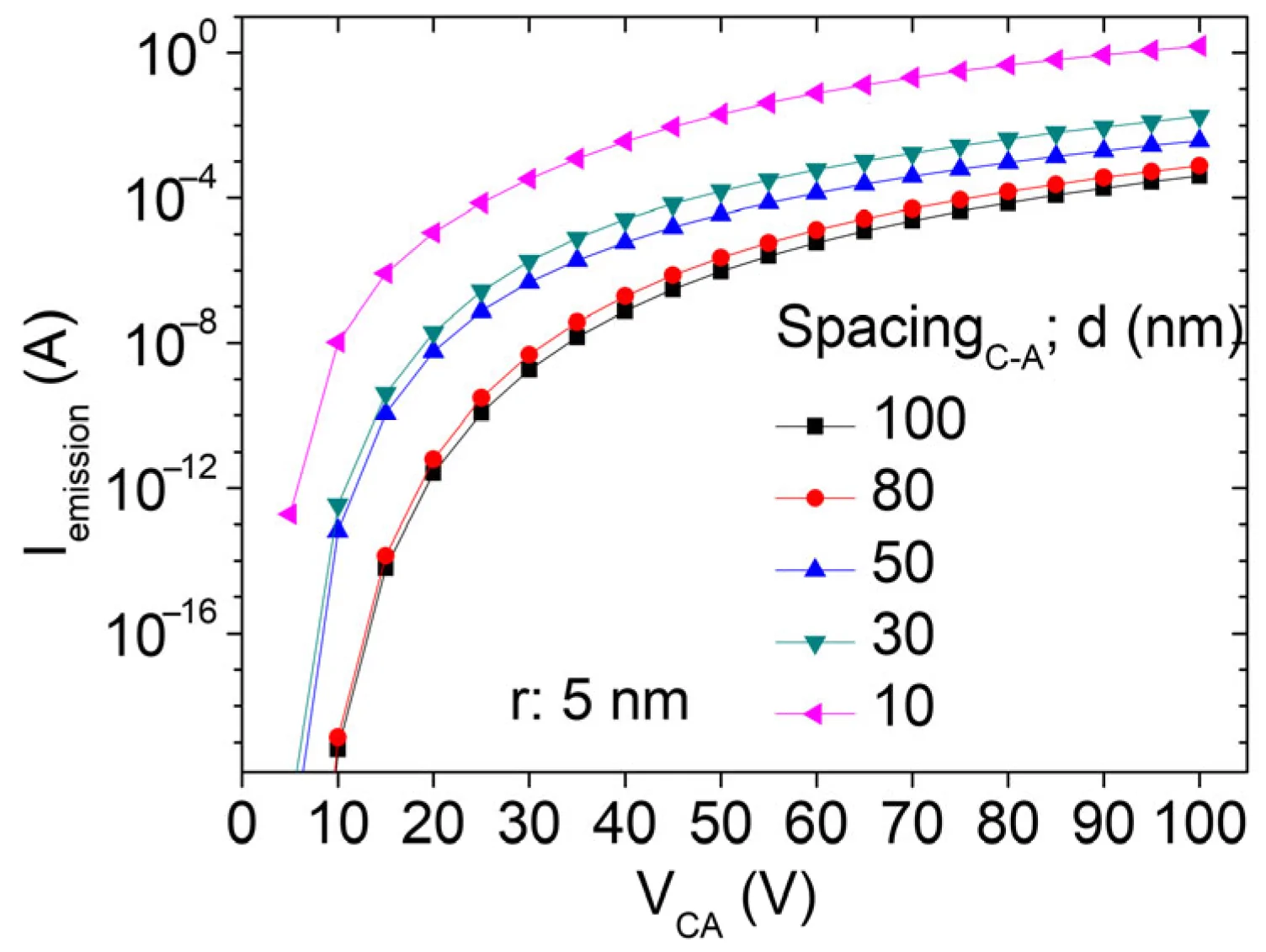
Simulated field I_emission_ as a function of applied voltage for tip-to-flat emitter-collector configurations, featuring cathode-to-anode spacings (*d*) of 100, 80, 50, 30, and 10 nm. All simulations assume a tip radius (r) of 5 nm.

Figure 14a–d illustrate simulated electron trajectories from the tip emitter to the planar counter-electrode for gap distances (d) of 30, 50, 80, and 100 nm, respectively. To ensure an objective comparison of this distance-dependent transport behavior, all particle trajectories were recorded at a fixed observation time, capturing electron velocities up to 10^7^ m/s. Decreasing the emitter-collector gap across all cases shifts electron transport toward higher velocities. At an ultra-short spacing of 10 nm, the intensely high electric field imparts an immense velocity to the electrons. This confirms that shorter distances facilitate both the initial excitation and the sustained motion of ultra-high-speed electrons. Furthermore, the cathode-to-anode spacing heavily impacts the simulated field-emission current (Figure 15). Increasing the gap spacing tenfold, from 10 nm to 100 nm, causes the I_CA_ to drop by nearly four orders of magnitude. Extrapolating this exponential decay from 10 to 100 nm simulations directly explains the sub-nanoampere currents observed in the enlarged experimental gaps, establishing a quantitative link between theoretical scaling and fabricated devices.

### 5.3. Effect of Tip Sharpness on Field Emission

To compare the effect of the curved tip in the C-FET structure and the triangular tip in the T-FET structure shown in Figure 2, three tip sharpness configurations, labeled t1, t2, and t3 (as defined in Figure 6), are analyzed. Among these, the sharpest emitter tip (t1) exhibits the lowest threshold voltage. For instance, it requires the smallest applied voltage to reach a field I_emission_ of 10 nA. Sharper tips also generate higher current at a given bias, reflecting the stronger local electric field concentration at the apex.

**Figure 16:**
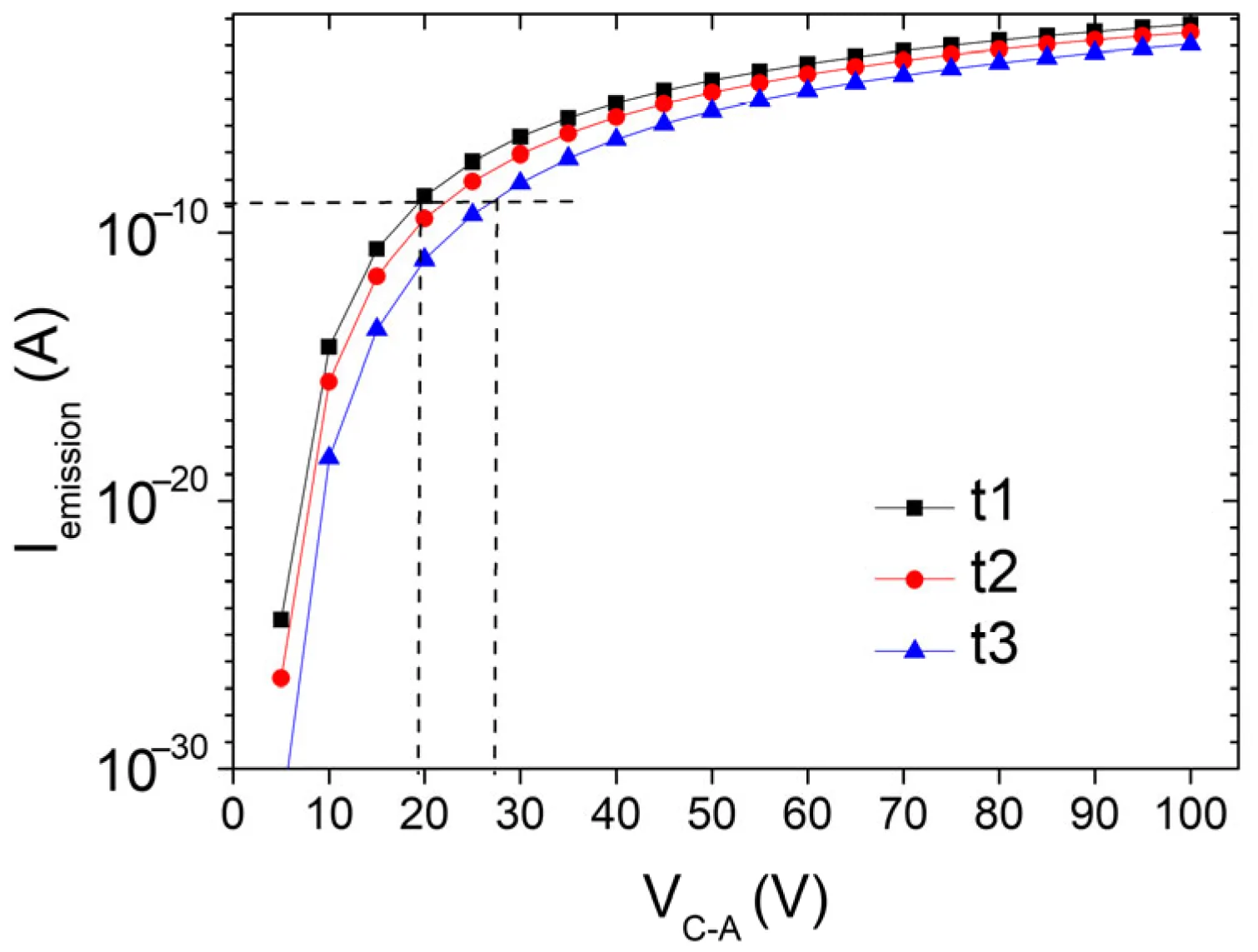
Simulated field I_emission_ as a function of applied voltage for the three tip sharpness configurations t1, t2, and t3 (defined in Figure 6), arranged in order of decreasing sharpness.

Figure 16 presents the simulated field emission versus applied voltage for the three tip sharpness configurations (t_1_, t_2_, and t_3_, as defined in Figure 6). These simulated trends match the experimental results in Figure 2, despite the C-FET maintaining a larger tip-to-anode spacing than the T-FET.This agreement confirms that tip sharpness is a key factor governing turn-on voltage and current efficiency in these field-emission triode structures. Note that the apparent slope moderation at high voltages in Figure 13, Figure 15 and Figure 16 is a visual artifact of the semi-logarithmic scale and the mathematical nature of the Fowler–Nordheim formulation, rather than physical saturation or numerical capping. Linear-scale evaluations confirm that I_emission_ continues to increase monotonically with voltage. Additionally, the figures show the simulated emission current integrated directly over the cathode apex surface. Under these two-terminal simulation conditions where gate interception is absent, the anode collection efficiency is taken as 100%, and thus I_CA_ is presented synonymously with I_emission_.

### 5.4. Electron Projection Under Gate Influence

**Figure 17:**
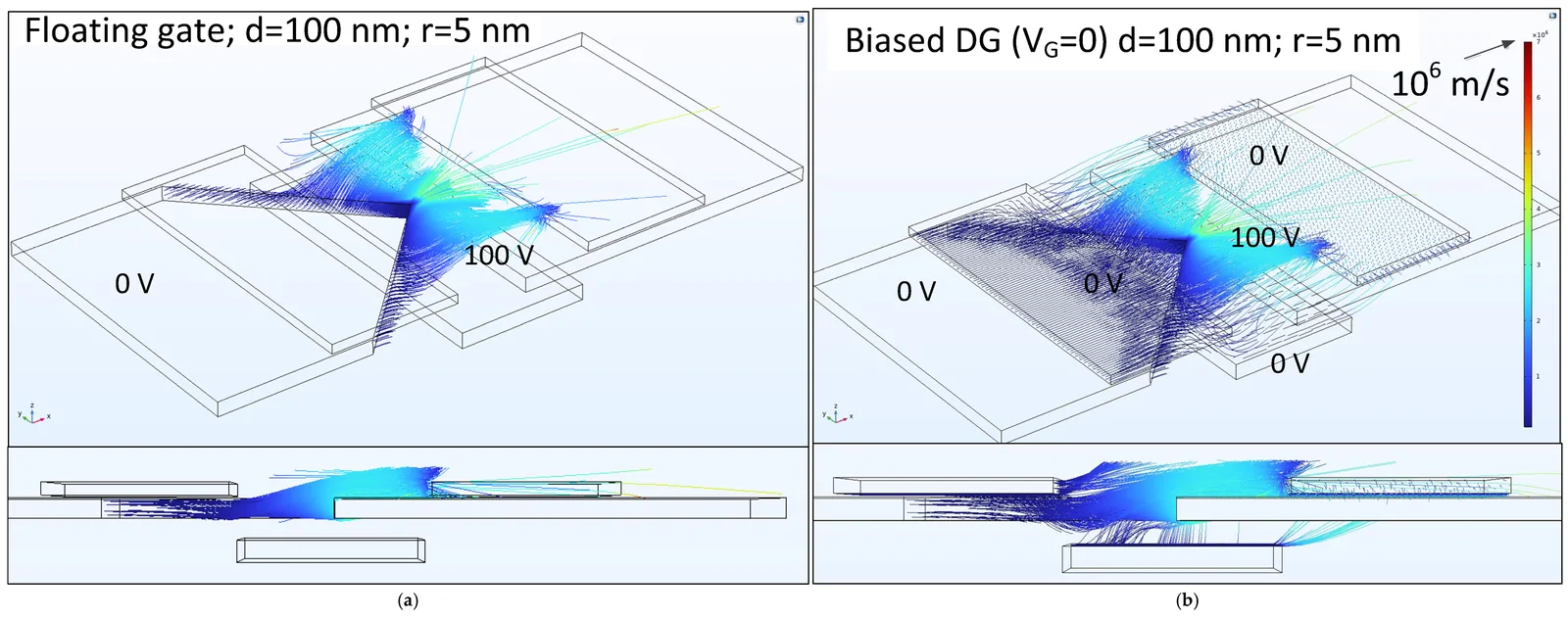
Simulated electron trajectories from the tip to the flat electrode (d = 100 nm, r = 5 nm) at 3 × 10^−12^ s for (**a**) a floating-gate configuration and (**b**) a biased-gate configuration.

To understand why biased-gate configurations produce higher electron emission than floating-gate arrangements, as observed in Figure 7a,b, we compare simulated electron trajectories under different gate conditions. Figure 17a,b present bird’s-eye views of electron paths originating from a sharp tip (radius r = 5 nm) and traversing a 100 nm air gap at t = 3 × 10^−12^ s for floating-gate and biased-gate configurations, respectively. Bottom insets in Figure 17a,b show the cathode-anode spatial relationship for top-gate (TG) and biased-gate (BG) geometries. In the floating-gate configuration, electron trajectories are primarily governed by the cathode–anode potential, resulting in a relatively narrow spatial distribution. When the gate is biased, the local electric field near the tip is strongly modified, leading to broader trajectory deflections toward the planar electrode, top gate (TG), and biased gate (BG).

**Figure 18:**
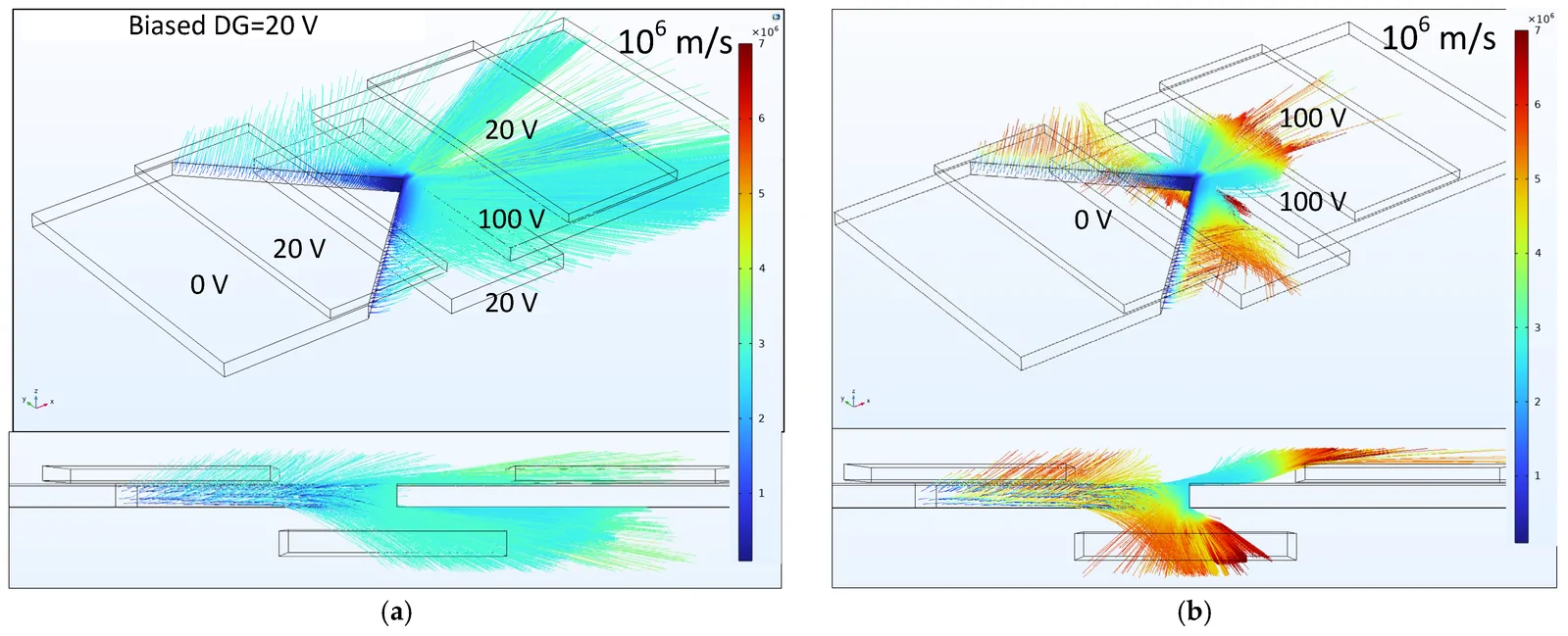
Simulated electron trajectories from the emitter tip to the planar electrode (d = 100 nm, r = 5 nm) at t = 3 × 10^−12^ s under a double-gate configuration with gate voltages of (**a**) 20 V and (**b**) 100 V.

Figure 18 further illustrates electron trajectories under a double-gate (DG) configuration for the same geometry (gap d = 100 nm, tip radius r = 5 nm) at t = 3 × 10^−12^ s, with gate voltages of (a) 20 V and (b) 100 V. At higher gate bias, the gate exerts greater control over the emitted electron flow, providing enhanced trajectory confinement. Increasing the gate voltage to 100 V significantly enhances field concentration near the tip, resulting in stronger beam divergence and more pronounced steering toward not only the anode but also the gate electrodes.

**Figure 19:**
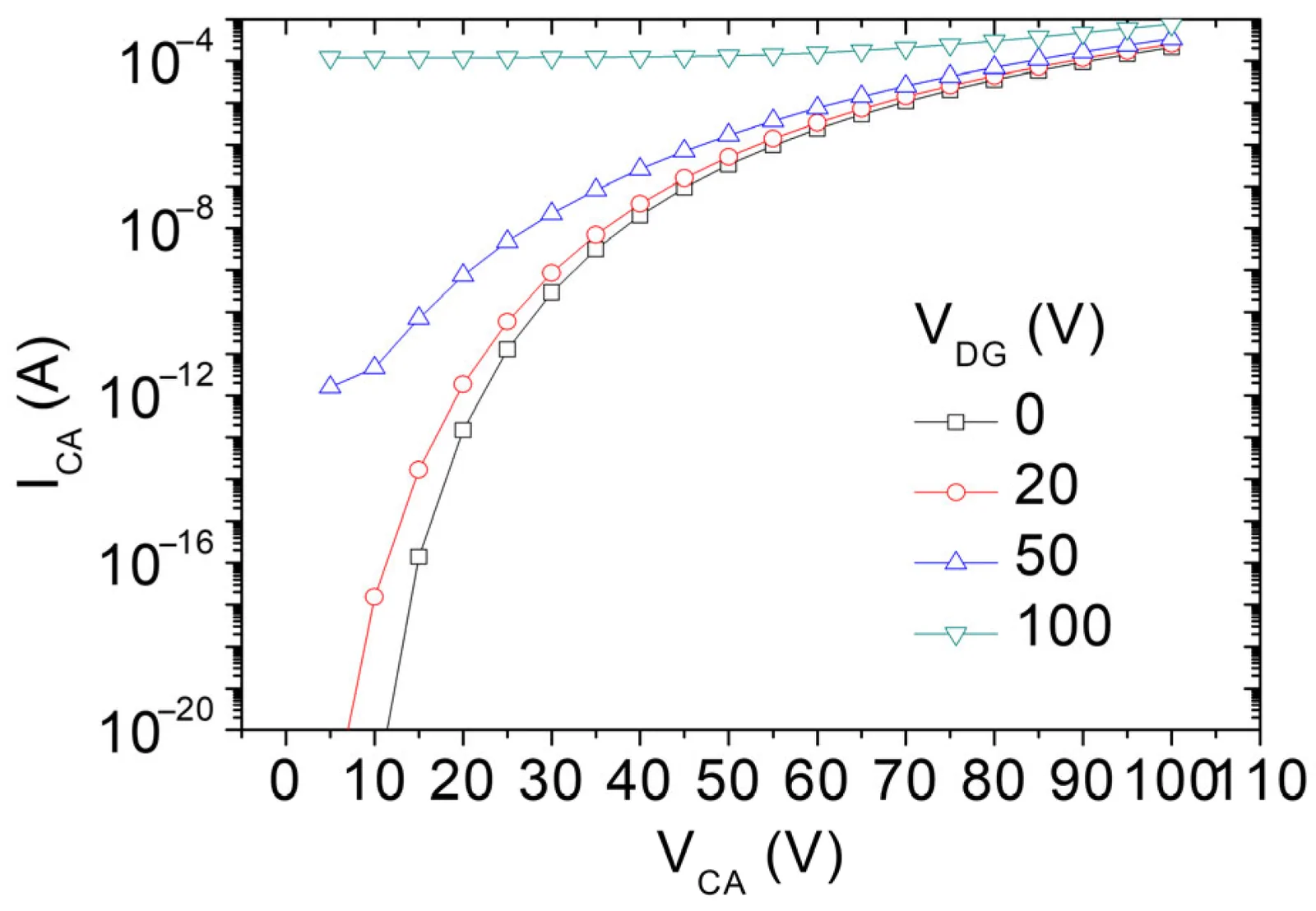
Simulated I_CA_ derived from electron trajectory simulations under a double-gate configuration with gate voltages (V_DG_) of 0, 20, 50, and 100 V.

**Figure 20:**
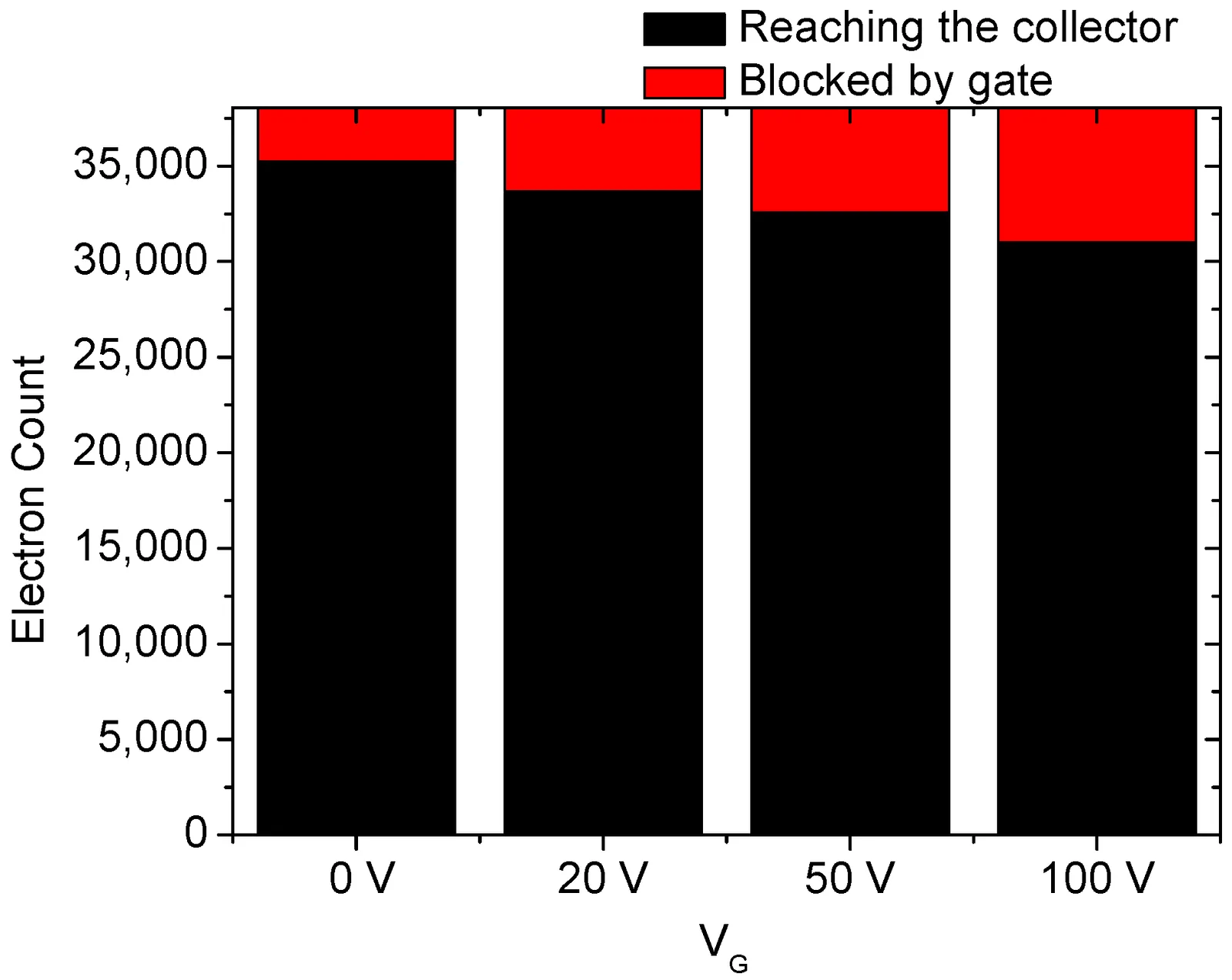
Simulated electron counts (totaling 39,460) reaching the collector (black) and blocked by the gate (red) under a double-gate configuration at V_DG_ of 0, 20, 50, and 100 V.

Figure 19 summarizes the simulated total I_CA_ derived from electron trajectory analysis for the emitter-to-anode system (d = 100 nm, r = 5 nm) at t = 3 × 10^−12^ s under a double-gate configuration with gate voltages (V_DG_) of 0, 20, 50, and 100 V. To evaluate the electrostatic beam-steering behavior and geometric interception probability shown in Figure 20, particle-tracing simulations were performed using a macro-particle ensemble totaling 39,460 test trajectories. While higher gate voltages increase the local electric field at the tip to boost I_emission_, the enhanced transverse field simultaneously deflects electron trajectories, increasing the geometric gate interception ratio (α = I_gate_/I_emission_) from 10% to 21%. This geometric tracking provides a practical first-order approximation for the net collected collector current (I_CA_ = I_emission_ − I_gate_) under purely electrostatically guided trajectories. Although the dual-gate design actively steers electron flow, this current interception mechanism alone remains insufficient to achieve a complete current cutoff.

## 6. Conclusions

This study demonstrates monolithically integrated dual-gate (DG) field-emission triodes fabricated in a standard 0.35 μm CMOS-MEMS process. Compared to single-gate baselines, the staggered DG structure lowers the threshold voltage by ~70% (from ~38 V to ~10 V), enhances low-voltage I_CA_ by over one order of magnitude, and increases g_d_ to ~17 pS (a 3.4-fold boost over single bottom gates), supported by peak simulated apex fields of ~6.8   10^9^ V/m. While high-frequency performance, radiation resistance, and long-term reliability warrant future circuit-level investigation, this work establishes essential design rules for on-chip gated vacuum microelectronic electron sources.

## Figures and Tables

**Table 1:** Parameters and symbols used in the Fowler–Nordheim analysis.

Name	Expression/Value	Unit	Description
$F_{local}$	Extracted from simulation	V/m	Local barrier field
$f_{val}$	$1.439965\times 10^{-9}\times \frac{F_{local}}{\emptyset^{2}}$	N/A	FN scaled variable
ν	$1-f_{val}+\frac{1}{6}f_{val}ln(f_{val})$	N/A	Correction factor
Ø	4.28	eV	Work function
V	10–100	V	Scan voltage

**Table 2:** Linear fitting parameters and performance metrics for electrostatic modulation efficiency across different gate configurations.

Gate Configuration	Slope at V_G_ = 0 V (S)	R^2^ (Goodness of Fit)	Primary Role in Modulation
TG	−19.78 ± 0.41 V	>0.989	Strong local field control (d = 37 nm)
BG	−12.04 ± 0.33 V	>0.986	Background field tuning (d = 290 nm)
DG	−25.31 ± 0.48 V	>0.995	Maximum electrostatic modulation

## Data Availability

The dataset is provided within the Word document and can be accessed using Origin or OriginPro 8.
